# The suppressive effect of the three-herb extract mixture on vascular and liver inflammation in atherogenic diet with high fructose-fed mice

**DOI:** 10.1080/13880209.2017.1412468

**Published:** 2018-05-18

**Authors:** Hae Seong Song, Hyun Jung Koo, Bong Kyun Park, Jeong Eun Kwon, Seon-A Jang, Hyun Jin Baek, Se Young Kim, Sung Ryul Lee, Se Chan Kang

**Affiliations:** a Department of Oriental Medicine Biotechnology, College of Life Sciences, Kyung Hee University, Yongin, Republic of Korea;; b Department of Medicinal and Industrial Crops, Korea National College of Agriculture and Fisheries, Jeonju, Republic of Korea;; c Department of Integrated Biomedical Science, Cardiovascular and Metabolic Disease Center, College of Medicine, Inje University, Busan, Republic of Korea

**Keywords:** Inflammation, fat accumulation, lipid-lowering, obesity, synergistic effect, P-selectin, vascular cell adhesion molecule-1

## Abstract

**Context:***Cynanchum wilfordii* (Maximowicz) Hemsley (Apocynaceae), *Arctium lappa* L. var. *rubescens* Frivald (Asteraceae) and *Dioscorea opposite* Thunb (Dioscoreaceae) root extracts have been widely used as an alternative for intervening obesity.

**Objectives:** The synergistic effect of three-herb mixture of *C. wilfordii*, *A. lappa* and *D. opposita* was determined on aortic and liver inflammatory responses.

**Materials and methods:** CWE, ALE and DOE were prepared from the root of *C. wilfordii*, *A. lappa* and *D. opposite* by 70% ethanol extraction, respectively. CADE was prepared using a powder mixture of 2 CWE:1 ALE:1 DOE. C57BL/6 mice were fed an atherogenic diet combined with 10% fructose (ATHFR) in the presence of 200 mg/kg/day CWE, ALE, DOE or CADE for 8 weeks (each group, *n* = 6). Biochemical profiles, protein expression of vascular cell adhesion molecule-1 (VCAM-1) on the aorta and liver were determined.

**Results:** CADE could significantly suppress the protein expression of VCAM-1 in both the aorta and liver (80% reduction) compared to ATHFR-fed mice. Impairment of liver function was significantly ameliorated by CADE supplement, as determined by GOT (60% reduction) and GPT (51% reduction) levels.

**Conclusions:** CADE should be considered when developing medications to suppress the vascular and liver inflammatory responses for individuals who are either non-responsive or resistant to lipid-lowering drugs.

## Introduction

Obesity is associated with low-grade chronic inflammatory responses involving excessive adipose tissue (Hotamisligil et al. 1993). Elevated production of proinflammatory cytokines such as tumour necrosis factor-α (TNF-α), interleukin-6 (IL-6), chemokines and coagulation proteins mediates multiple processes including metabolic and vascular alterations in the body (Hotamisligil et al. 1993; Ferrante 2007; Fernandez-Sanchez et al. 2011). Inflammatory processes arising from metabolic abnormalities are known to precipitate the development of cardiovascular diseases (CVDs), which are major causes of morbidity and mortality worldwide. Abnormally high levels of cholesterol and triglycerides (TGs) in the blood contribute to the initiation of vascular inflammation causing atherosclerosis and nonalcoholic fatty liver disease (NAFLD), which is characterized by hepatic lipid accumulation (Demir et al. 2015). Lipid-lowering drugs and lifestyle modifications aiming at weight-loss remain the mainstay of treating obesity and to delay or suppress obesity related pathologies. Unfortunately, long-term use of anti-obesity drugs is limited by their high attrition rates and lack of long-term morbidity and mortality data (Padwal and Majumdar 2007). In addition, anti-obesity drugs are limited by modest efficacy and low rates of persistence with treatment. For example, statins are class of hydroxymethylglutaryl-coenzyme A (HMG-CoA) reductase inhibitors and are prescribed as low-density lipoprotein cholesterol (LDL-C)-lowering agents with the best clinical trial evidence of CVD outcome benefits and are the first-line therapy for hypercholesterolemia (Kearney et al. 2008). However, statins may not be tolerated by all patients in doses large enough to attain LDL-C goals (Preiss et al. 2011), and sometimes persons at heightened risk for CVD will require binary or ternary regimens involving statins in concert with niacin, fibric-acid derivatives or bile acid resins (Jacobson 2001). Moreover, it has been found that statins may be associated with increased risk (particularly at high doses) of new-onset diabetes and unfavourable glycaemic effects (Preiss et al. 2011). In addition to heritable conditions such as a familial hypercholesterolemia and familial combined hyperlipidaemia, some patients fail to achieve favourable LDL-C control even under this medical therapy (Kataoka et al. 2015) and, thus, are predisposed to higher risk of atheroma progression (Kataoka et al. 2015). NAFLD has been associated with several cardiovascular (CV) risk factors including obesity, dyslipidaemia, hyperglycaemia, hypertension and smoking (Demir et al. 2015). NAFLD is also characterized by atherogenic dyslipidaemia, postprandial lipaemia and high-density lipoprotein (HDL) dysfunction. Most importantly, NAFLD patients are at increased risk for both liver and CVD morbidity and mortality (Katsiki et al. 2016). Those patients who are non-responders to statin or lipid-lowering therapies may be predisposed to higher risk of atheroma progression even in the process of finding a best interventional choice. Phytochemical based interventions that are effective in suppressing vascular inflammation, irrespective of their strong lipid-lowering potential provide an alternative choice for treatment.

Synergistic interactions between the components of individual or mixtures of herbs have been suggested, since positive and negative aspects of interactions cannot be explained by an isolated single active ingredient, the efficacy of apparently low doses of active constituents in a herbal product, and/or arithmetic summation of the actions of individual components (Williamson 2001; Gilbert and Alves 2003; Periago et al. 2004; Hannan et al. 2016). Optimal dose blending of plant extracts possessing lipid-lowering activity and alleviating inflammatory reactions is helpful in minimizing adverse events along with maximizing beneficial outcomes. To this aim, we selected three herbal extracts, *Cynanchum wilfordii* (Maximowicz) Hemsley (Apocynaceae) (Jang et al. 2016), *Arctium lappa* L. vari. rubescens Frivald (Asteraceae) (Lee et al. 2012) and *Dioscorea opposite* Thunb (Dioscoreaceae) (Cho et al. 2016) roots, which have been widely used as alternatives for treating obesity. In this study, we determined the optimal formulation using *C. wilfordii*, *A*. *lappa* and *D*. *opposita* for suppressing mRNA expression levels of ICAM-1 and VACM-1 in HASMC cells. Using the formulation of 2 parts *C. wilfordii*; 1 part *A*. *lappa*; 1 part *D*. *opposita* (CADE), we determined the effects of CADE on changes in the lipid profile and expression of VCAM-1 and P-selectin in the arteries of the ATHFR mouse model. In addition, the effect of CADE on changes in liver function, fat accumulation and inflammatory responses in the liver was assessed.

## Materials and methods

### Materials and reagents

Diethyl ether, Oil red O, simvastatin (Simv), insulin, dexamethasone, 3-isobutyl-1-methylxanthine (IBMX), TNF-α and 3-(4,5-dimethylthiazol-2-yl)-2,5-diphenyltetrazolium bromide (MTT) were obtained from Sigma (St. Louis, MO). Omega-3 was purchased from GC Rieber (AS, Kristiansand, Norway). Antibodies against vascular cell adhesion molecule-1 (VCAM-1) and P-selectin were obtained from Santa Cruz Biotechnology (Santa Cruz, CA). AdipoRed^TM^ assay reagent was purchased from Lonza (Walkersville, MD).

### Preparation of ethanol extracts of herbal samples

Roots from *C. wilfordii*, *A. lappa* L. and *D. opposita* were collected from Jeju (Korea) or Jecheon (ChungBuk, Korea) in November 2015. All raw herbal samples were confirmed by Prof. Se Young Kim (Kyung Hee University, Korea) and authenticated based on their microscopic and macroscopic characteristics. A voucher specimen (MPRBP00962, NMR114, NMR256) of each herb was deposited at the department of Oriental Medicine Biotechnology, Gachon University.

The dried roots of *C*. *wilfordii* (25 kg), *A*. *lappa* (12.5 kg) and *D*. *opposita* (12.5 kg) were extracted with 70% EtOH at 82 ± 2 °C for 24 h and filtered. The ethanol extracts were concentrated using a rotary evaporator and were then lyophilized. Final product yields were 4.1 kg *C*. *wilfordii*, 2.0 kg *A*. *lappa* and 1.3 kg *D*. *opposita*, and the three-herb mixture was composed of the three extracts at the ratio of 2:1:1, respectively. Here, the mixing ratio among three herbs was chosen based on its maximal inhibitory effect on the protein expression level of VCAM-1 shown in a previous report (Cho et al. 2016). All samples were stored at 4 °C until use.

### Cell culture

Human aortic smooth muscle cells (HASMCs) were obtained from ScienCell Research Laboratories (San Diego, CA) and were cultured in smooth muscle cell medium (SMCM, ScienCell Research Laboratories, Carlsbad, CA) supplemented with 2% foetal bovine serum (FBS), 1% smooth muscle cell growth supplement (SMCGS) and 1% penicillin/streptomycin. 3T3-L1 (ATCC, Manassas, VA) cells were cultured Dulbecco’s modified eagle’s medium (DMEM, Invitrogen, Carlsbad, CA) supplemented with 10% BCS, penicillin/streptomycin in a humidified incubator at 37 °C and 5% CO_2_.

### MTT assay

HASMCs were seeded at a density of 1 × 10^5^ cells/mL in 96-well plates. Cells were treated with the samples for 24 h and then incubated with 10 μL of 5 mg/mL MTT (Sigma, St. Louis, MO) for 4 h. The supernatant was then removed, after which the formazan was dissolved in 100 μL of DMSO with a shaker for 10 min. The optical density was measured at a 570 nm wavelength using a Multi-Reader instrument (TECAN, Zurich, Switzerland).

### Quantitative real-time PCR

Total RNA from HASMCs was isolated with Trizol reagent (Invitrogen, Carlsbad, CA). Quantity and purity of the RNA were identified by measuring the absorbance values at 260 nm and ratio of 260–280 nm, respectively. Next, 3 μg of total RNA were transcribed by using the PrimeScriptII 1st strand cDNA Synthesis kit (Takara, Tokyo, Japan). Quantitative real-time polymerase chain reactions (qRT-PCRs) were performed with a MX3005P (Stratagene, La Jolla, CA) using the following primer pairs: 5′-ATTTTCTGGGGCAGGAAGTT-3′ and 5′-ACGTCAGAACAACCGAATCC-3′ for human VCAM-1; 5′-AGCACCTCCCCACCTACTTT-3′ and 5′-AGCTTGCACGACCCTTCTAA-3′ for human ICAM-1; 5′-AACTTTGGCATTGTGGAAGG-3′ and 5′-ACACATGGGGGTAGGAACA-3′ for human glyceraldehydes-3-phophate dehydrogenase (GAPDH). SYBR Premix Ex Taq II (Takara, Tokyo, Japan) was used for real-time PCR. The final volume of the mixture was 25 μL and contained 2 μL cDNA template, 12.5 μL master mix, 1 μL each primer (10 μM stock solution) and 8.5 μL sterile distilled water. The thermal cycling profile consisted of a pre-incubation step at 95 °C for 10 min, followed by 40 cycles at 95 °C (30 s), 55 °C (60 s) and 72 °C (30 s). Relative quantitative evaluation of VCAM-1 and ICAM-1 was performed using comparative CT (cycle threshold) (Livak and Schmittgen 2001).

### Experimental animals

The experimental animal facility and study were approved by the Animal Care and Use Committee of Gachon University (GIACUC-R2015008). All experimental procedures were compliant with the Guide for the Care and Use of Laboratory Animals (National Institutes of Health, Bethesda, MD) and the National Animal Welfare Law of the Republic of Korea.

Atherogenic diet (D12336; Research Diets Inc., New Brunswick, NJ) contains 45% carbohydrate, 25% protein and 35% fat, whereas chow diet (2018S Teklad Global 18% Protein Rodent diet; Envigo, Madison, WI) contains approximately 57.3% carbohydrate, 18.9% protein, 5.7% crude oil and others (Jang et al. 2016). High fructose intake is associated with high incidence of CVD in human (Dietze et al. 1976; Aeberli et al. 2007; Tonstad et al. 2007), it is used to induce vascular inflammation in mouse and rat model (Dai and McNeill 1995; Jang et al. 2016). Six-week-old male C57BL/6 mice were obtained from Korea DBL Inc. (Chungbuk, Korea) and maintained in a controlled environment of 22 ± 2 °C and 55 ± 5% humidity with 12 h light-dark cycle for 1 week prior to the experiments. Mice were randomly divided into eight groups (each *n* = 6). Mice fed a normal chow diet were grouped as a negative control group (NC). Other mice were fed an atherogenic diet combined with 10% fructose in their drinking water (ATHFR) in the presence or absence of CWE (200 mg/kg/day), ALE (200 mg/kg/day), DOE (200 mg/kg/day), CADE (200 mg/kg/day), omega-3 (500 mg/kg/day) or simvastatin (10 mg/kg/day). Previously, CWE at the dose of 200 mg/kg could significantly suppress both high fat-induced liver damage (Jang et al. 2016) and vascular inflammation. Thus, treating dose of ALE, DOE and CADE was chosen at a same dose of CWE to evaluate whether synergistic effect could be produced when three herbs were combined. All supplements were delivered six days per week with oral gavage for 8 weeks. An appropriate dosing volume of saline or extract was determined after weighing the animal daily. Intragastric delivery of saline or extract six days per week was carefully performed by a well-trained researcher to minimize animal stress. Clinical signs and general appearances were observed during the experimental period. At the end of experiment, mice were euthanized by diethyl ether.

### Haematoxylin and eosin (H&E) staining

Mice liver and aorta were fixed with 4% formaldehyde for two days, dehydrated, and embedded in paraffin. Thin sections were placed on glass slides, dewaxed, and rehydrated with PBS. Subsequently, sections were stained with H&E. After staining, the images were viewed under microscopy with the Olympus DP controller software program (Tokyo, Japan). For the liver, the number and area of macrovascular and microvascular steatosis were calculated and plotted. For measurement of aorta thickness, images were focused on areas chosen randomly. The average of aortic thickness was measured by DP controller, and we show the best quality images and quantitative graphs.

### Immunohistochemistry

Immunohistochemical analysis was performed as described previously (Zheng et al. 2009). Briefly, mouse liver and aorta were fixed with 4% formaldehyde for two days, dehydrated, and embedded in paraffin. Thin sections were placed on glass slides, deparaffinized in xylene and ethanol, and rehydrated in PBS. Paraffin sections were incubated with anti-VCAM-1 antibody (1:100) or anti-P-selectin antibody (1:100). Sections were subsequently incubated with peroxidase-conjugated secondary antibodies. Data are the average of four independent counts per section.

### Oil red O staining

Frozen liver sections were washed with phosphate-buffered saline (PBS), fixed in 4% formalin, and embedded in paraffin. Sections were stained with 0.5% oil red O (Sigma-Aldrich, St. Louis, MO) and the red lipid droplets were visualized by microscopy. For quantitative analysis, the total area of red pixels on the oil red O-stained sections was measured using Image J software program (National Institutes of Health, Rockville, MD).

### Determination of lipid content in 3T3-L3 adipocytes

To induce adipocyte differentiation, confluent 3T3-L1 (designated day 0) were incubated with DMEM involving 10% FBS, 10 μg/mL insulin, 1 μM dexamethasone and 0.5 mM IBMX for two days. Afterwards, cells were fed DMEM containing 10% FBS and 10 μg/mL insulin for two days. Finally, cells were incubated using DMEM combined with 10% FBS every other day. Eight days after differentiation, cells washed twice with PBS, fixed with 4% formaldehyde at room temperature for 5 h, and stained with AdipoRed^TM^ assay reagent. For identification, representative images were measured under fluorescence microscopy (OLYMPUS, Tokyo, Japan) at 488 nm.

### Statistical analysis

Data are expressed as mean ± SEM. Results were subjected to an analysis of variance (ANOVA) using the Tukey test. Significant values are indicated by a superscript (*p < 0.05 compared with the NC group; #*p* < 0.05 compared with the ATHFR group).

## Results

### Effect of CADE on the mRNA expression of VCAM-1 and ICAM-1 in HASMC cells

**Figure 1.**
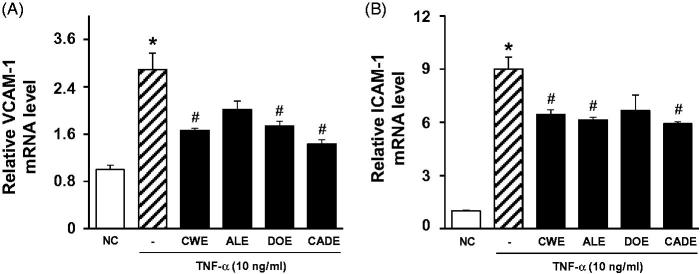
The suppressive effects of CWE, ALE, DOE and CADE on TNF-α induced mRNA expression level of VCAM-1 (A) and ICAM-1 (B). HASMC cells were treated with 20 μg/mL of CWE, ALE, DOE and CADE in the presence of TNF-α (10 ng/mL) for 12 h. The mRNA expression levels of VCAM-1 and ICAM-1 were determined by RT-PCR. Data are mean ± SEM. *p < 0.05, significantly different from the untreated group, #*p* < 0.05, significantly different from the TNF-α group. ATHFR: an atherogenic diet plus 10% fructose; CWE: *C. wilfordii* extract; ALE: *A. lappa* L. extract; DOE: *D. opposita* extract; CADE: mixture of CWE:ALE:DOE at a ratio of 2:1:1; NC: untreated control.

When the cytotoxicities of CWE, ALE, DOE and CADE were tested on HASMC cells through MTT reduction, there was no significant cytotoxicity at the 100 μg/mL dose after 24 h for any of the tested herbal extracts (data not shown). One of the initial and key events in the endothelium response to inflammatory stimuli is the expression of cell adhesion molecules (CAMs) such as VCAM-1, ICAM-1 and P-selectin (Petruzzelli et al. 1999; Hansson 2005). To be considered as beneficial for alleviating inflammatory responses in the endothelium, tested extracts should be involved in the suppression of gene expression stimulated by proinflammatory cytokines such as a TNF-α. Thus, the suppressive effects of CWE, ALE, DOE, and their combined mixture at a 2:1:1 ratio (CADE) on the mRNA expression level of VCAM-1 and ICAM-1 in HASMC cells were determined in the presence of TNF-α (10 ng/mL). Among the three different isolated extracts, CWE was the most effective in suppression of both VCAM-1 (Figure 1(A)) and ICAM-1 (Figure 1(B)). ALE was only effective in the suppression of ICAM-1 mRNA expression, whereas DOE was only effective in the suppression of VCAM-1 mRNA expression. The suppressive effects of CADE on the mRNA expression levels of VACM-1 and ICAM-1 were either equal or superior to CWE alone, because the overall content of CWE in CADE is half that of the CWE alone group. This result suggests that CADE could suppress the vascular inflammatory responses associated with increased VCAM-1 and/or ICAM-1 without loss of the strong potential of CWE even at a half dose.

### Effect of CADE on adipogenesis in 3T3-L1 preadipocytes

**Figure 2.**
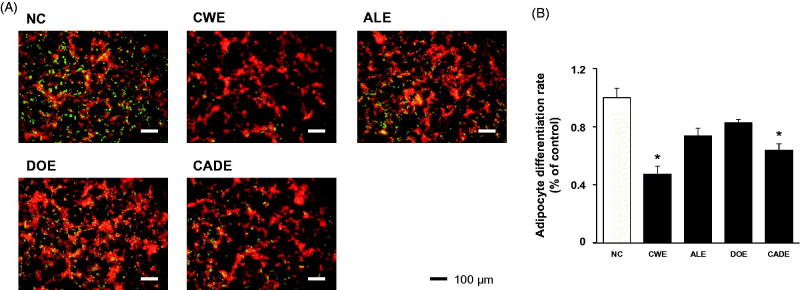
The suppressive effects of CWE, ALE, DOE and CADE on adipogenesis in 3T3-L1 cells. 3T3-L1 preadipocytes were differentiated in the presence or absence of CWE, ALE, DOE and CADE (200 μg/mL). After staining with AdipoRed^TM^, the lipid droplets were quantified by the intensities of fluorescence. Small dot indicates a lipid droplet. Original magnification, ×100. Data are mean ± SEM. *p < 0.05, significantly different from the untreated group. CWE: *C. wilfordii* extract; ALE: *A. lappa* L. extract; DOE: *D. opposita* extract; CADE: mixture of CWE:ALE:DOE at ratio of 2:1:1; NC: untreated control.

Adipocytes are widely used to study pre-adipocyte differentiation, and adipogenesis plays a key role in the development of adult obesity (Ali et al. 2013). Using the 3T3-L1 preadipocytes, we determined the adipogenesis suppressing effects of CWE, ALE, DOE and CADE on the level of fat accumulation in differentiated 3T3-L1 cells (Figure 2(A,B)). Compared to the untreated control, CWE treatment highly inhibited the accumulation of lipid in the cells, and both ALE and DOE treatment were less effective than CWE treatment. The inhibitory effect of CADE on lipid accumulation in 3T3-L1 cells was stronger than that of either ALE or DOE, but was less potent than that of CWE (Figure 2(A,B)).

### Changes in body weight, food intake and water intake

**Figure 3.**
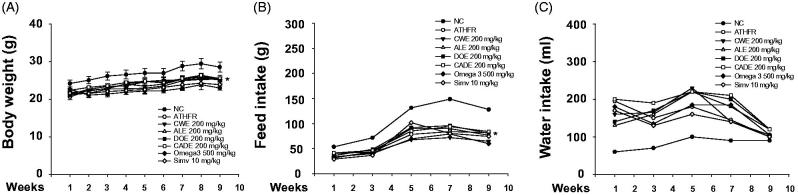
Changes in body weight and both food and water intake rate. Mice were fed a normal chow diet or an ATHFR diet in the presence or absence of CWE, ALE, DOE and CADE for 8 weeks. Body weight and food and water intake were measured weekly. Data are mean ± SEM (each group, *n* = 6). ATHFR: an atherogenic diet plus 10% fructose; CWE: *C. wilfordii* extract; ALE: *A. lappa* L. extract; DOE: *D. opposita* extract; CADE: mixture of CWE:ALE:DOE at ratio of 2:1:1; NC: untreated control.

To determine the lipid lowering-independent effect of CADE on ATHFR-mediated vascular and liver inflammatory responses, mice were fed with an ATHFR diet in the presence or absence of CWE, ALE, DOE and CADE at 200 mg/kg/day for 8 weeks. Clinical signs and general appearances were observed for five days a week. There were no treatment related symptoms or mortality during the experimental course and at any dose given. ATHFR fed mice showed decreases in body weight and food intake rate but increases in the water intake rate (Figure 3). There was no significant difference among ATHFR fed mice irrespective of the kind of supplement given.

### Effect of CWE, ALE, DOE and CADE on changes in liver, kidney, spleen and thymus weight

**Figure 4.**
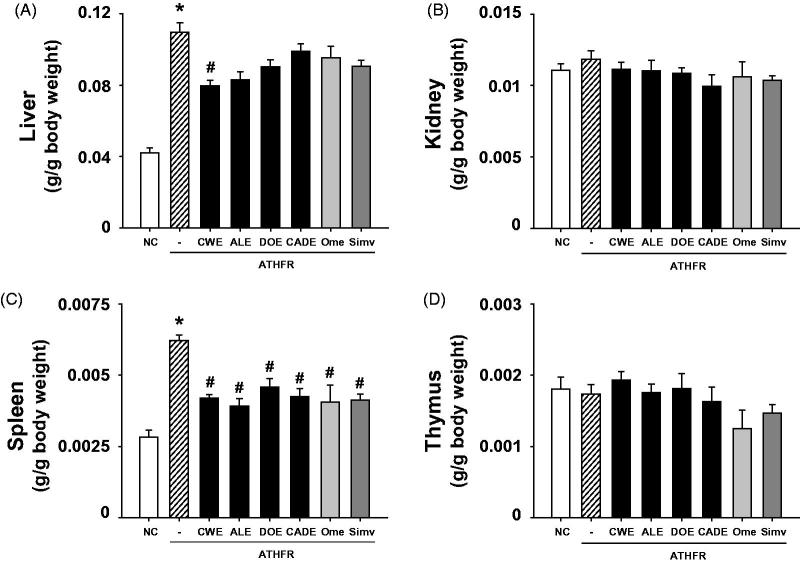
Changes in the weight of liver, kidney, spleen and thymus. Mice were fed with normal chow diet and ATHFR diet in the presence or absence of CWE, ALE, DOE and CADE for 8 weeks. At the end of experiment, the weights of liver, kidney, spleen and thymus were measured. Data are mean ± SEM (each group, *n* = 6). Omega-3 and simvastatin were used as controls for nutritional alternative and lipid-lowering drug, respectively. *p < 0.05, significantly different from the NC group, #*p* < 0.05, significantly different from the ATHFR group. ATHFR: an atherogenic diet plus 10% fructose; CWE: *C. wilfordii* extract; ALE: *A. lappa* L. extract; DOE: *D. opposita* extract; CADE: mixture of CWE:ALE:DOE at a ratio of 2:1:1; NC: untreated control.

The ATHFR diet causes hepatic hypertrophy and fibrosis resulting from the accumulation of fat and lipid in liver (Kitamori et al. 2012). Splenomegaly can be induced by impairment of liver function (da Silva et al. 2012). Changes in organ weight may be associated with metabolic distress and/or low-grade inflammation. Liver, kidney, spleen and thymus were collected at the end of our experiment and were weighed (Figure 4). The weights of the kidneys and thymuses from the ATHFR group were not different from those in the normal chow-diet group, but the weights of the livers and spleens were highly increased in animals after 8 weeks on the ATHFR diet as compared with animals in the normal chow diet group. The reduction in liver weight was only observed after CWE supplementation, but all four supplements were highly effective in reducing spleen weight. Interestingly, CADE, which contains half of the concentration of CWE as the CWE alone group, showed a similar reduction in spleen weight increase.

### Effect of CWE, ALE, DOE and CADE on the status of lipid profiles

**Figure 5.**
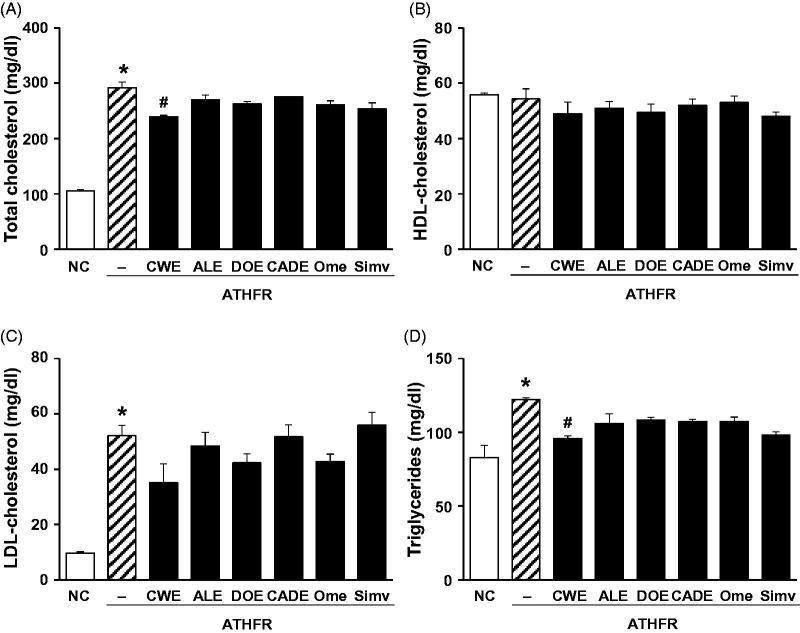
Effect of CWE, ALE, DOE and CADE on the status of lipid profiles. Mice were fed with normal chow diet or ATHFR diet in the presence or absence of CWE, ALE, DOE and CADE for 8 weeks. At the end of experiment, blood specimens were collected the serum level of total-cholesterol (A), HDL-cholesterol (B), LDL-cholesterol (C), and triglyceride levels (D) were measured using commercially available assay kits. Data are mean ± SEM (each group, *n* = 6). Omega-3 and simvastatin were used as controls for nutritional alternative and lipid-lowering drug, respectively. ATHFR: an atherogenic diet plus 10% fructose; CWE: *C. wilfordii* extract; ALE: *A. lappa* L. extract; DOE: *D. opposita* extract; CADE: mixture of CWE:ALE:DOE at a ratio of 2:1:1; NC: untreated control.

Total cholesterol (T-C), LDL-cholesterol (LDL-C) and TG were markedly increased in ATHFR diet-fed mice, but HDL-cholesterol (HDL-C) was unchanged (Figure 5). Among the tested supplements, CWE was significantly effective in lowering the T-C (15% reduction) and TG (20% reduction). However, the single extracts of ALE or DOE were less potent than CWE. CADE containing half of the CWE concentration as the CWE alone supplement was also less potent than that of CWE alone. CADE showed a suppressing effect on T-C, LDL-C and TG but without statistical significance. This result suggests that a blood lipid lowering effect is strongly present in CWE, and, thus, the lower concentration of CWE in CADE resulted in less effective reduction of lipid and cholesterol levels than that of CWE alone.

### Effect of CWE, ALE, DOE and CADE on the ATHFR diet-induced thickening of the aorta

**Figure 6.**
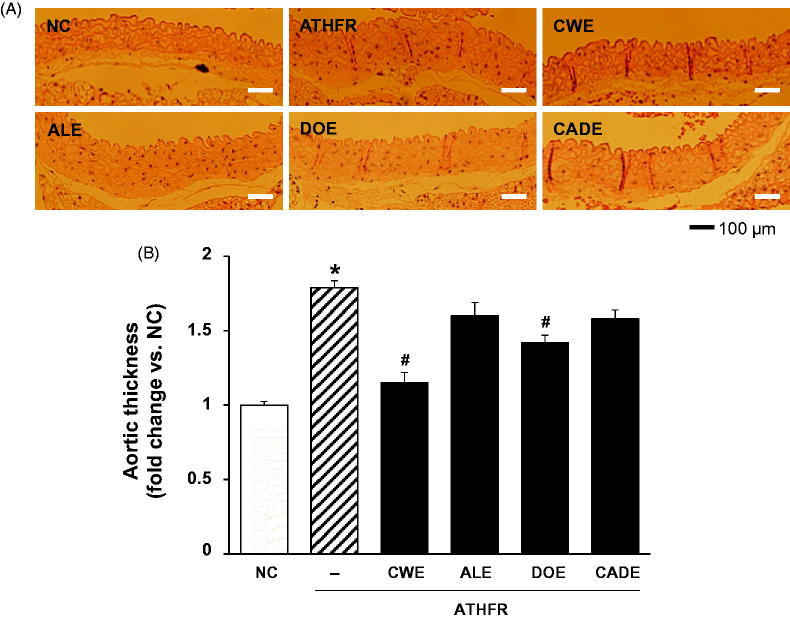
Effect of CWE, ALE, DOE and CADE on the thickening of the aorta in ATHFR diet-fed mice. Mice were fed with a normal chow diet or ATHFR diet in the presence or absence of CWE, ALE, DOE and CADE for 8 weeks (each *n* = 6). At the end of the experiment, aorta tissues were collected and stained with H/E staining. Data are mean ± SEM (each group, *n* = 6). Omega-3 and simvastatin were used as controls for nutritional alternative and lipid-lowering drug, respectively. Original magnification, ×100. *p < 0.05, significantly different from the NC group, #*p* < 0.05, significantly different from the ATHFR group. ATHFR: an atherogenic diet plus 10% fructose; CWE: *C. wilfordii* extract; ALE: *A. lappa* L. extract; DOE: *D. opposita* extract; CADE: mixture of CWE:ALE:DOE at a ratio of 2:1:1; NC: untreated control.

High lipid content in the vasculature can cause vascular damage and result in thickening of the aorta. When the thickness of the aorta was determined among groups by H/E staining, animals in ATHFR diet-fed group showed greater than 1.7-fold thickening compared to normal chow-fed animals (Figure 6). Both CWE and DOE supplements alleviated the aorta thickening in ATHFR diet-fed mice, but CWE was more potent than that DOE. The thickening of the aorta was suppressed by CADE supplementation but without statistical significance. This less suppressive effect of CADE on aorta thickening might be associated with its decreased lipid-lowering effect as compared with that of CWE alone (Figure 5).

### Effects of CWE, ALE, DOE and CADE on protein expression levels of VCAM-1 and P-selectin in the aorta

**Figure 7.**
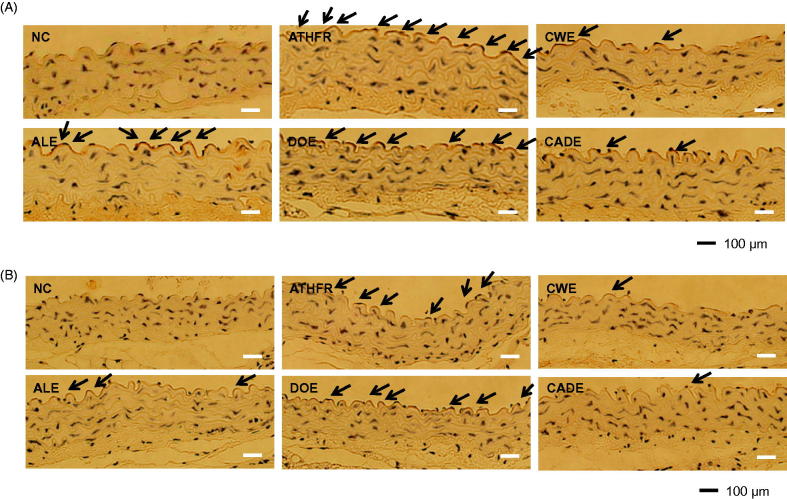
Effect of CWE, ALE, DOE and CADE on protein expression levels of VCAM-1 and P-selectin in the aorta from ATHFR diet-fed mice. Mice were fed with normal chow diet or ATHFR diet in the presence or absence of CWE, ALE, DOE and CADE for 8 weeks (each *n* = 6). At the end of experiment, aorta tissues were collected, and the protein expression levels of VCAM-1 (A) and P-selectin (B) were detected by immunohistochemistry. Omega-3 and simvastatin were used as controls for nutritional alternative and lipid-lowering drug, respectively. Original magnification, ×200. Bar indicates 100 μm. ATHFR: an atherogenic diet plus 10% fructose; CWE: *C. wilfordii* extract; ALE: *A. lappa* L. extract; DOE: *D. opposita* extract; CADE: mixture of CWE:ALE:DOE at a ratio of 2:1:1; NC: untreated control.

The increased expression level of VCAM-1 and P-selectin in the aorta may be associated with thickening of the aorta. When the protein expression levels of VCAM-1 (Figure 7(A)) and P-selectin (Figure 7(B)) were evaluated by immunohistochemistry, significantly increased expression levels of VCAM-1 and P-selectin were suppressed by CADE supplementation with stronger potency than that of either the ALE or DOE alone groups. The strong inhibitory effects of CWE on the expression of VCAM-1 and P-selectin seem to be related with its transcriptional regulatory role (Figure 7) and its lipid-lowering effects. The strong inhibitory effect of CADE on the expression of VCAM-1 (Figure 7) was also found in ATHFR-fed mice with better potency than that of ALE or DOE.

### Effect of CWE, ALE, DOE and CADE on liver function

**Figure 8.**
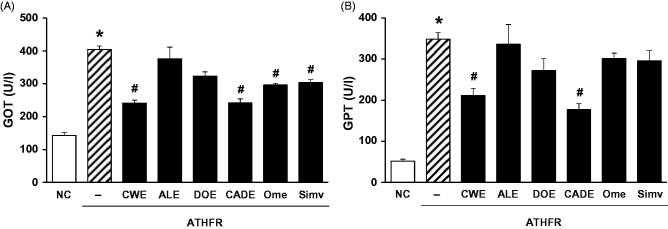
Effect of CWE, ALE, DOE and CADE on enzymatic activities of GOT and GPT in ATHFR diet-fed mice. Mice were fed with normal chow diet or an ATHFR diet in the presence or absence of CWE, ALE, DOE and CADE for 8 weeks (each *n* = 6). At the end of the experiment, both GOT and GPT levels were determined using a colorimetric method. Omega-3 and simvastatin were used as controls for nutritional alternative and lipid-lowering drug, respectively. *p < 0.05, significantly different from the NC group, #*p* < 0.05, significantly different from the ATHFR group. ATHFR: an atherogenic diet plus 10% fructose; CWE: *C. wilfordii* extract; ALE: *A. lappa* L. extract; DOE: *D. opposita* extract; CADE: mixture of CWE:ALE:DOE at a ratio of 2:1:1; NC: untreated control.

Liver weight was significantly increased by ATHFR diet (Figure 4). This enlargement of liver mass may be associated with deterioration of liver function. When the enzymatic activities of GOT and GPT were determined, significant increases of enzymatic activities of GOT (Figure 8(A)) and GPT (Figure 8(B)) were shown. Both omega-3 and Simv were only effective in suppressing GOT activity. CADE strongly suppressed the both GOT and GPT enzymatic levels and was comparable to that of CWE alone.

### Effect of CWE, ALE, DOE and CADE on lipid content and fat accumulation in the liver

**Figure 9.**
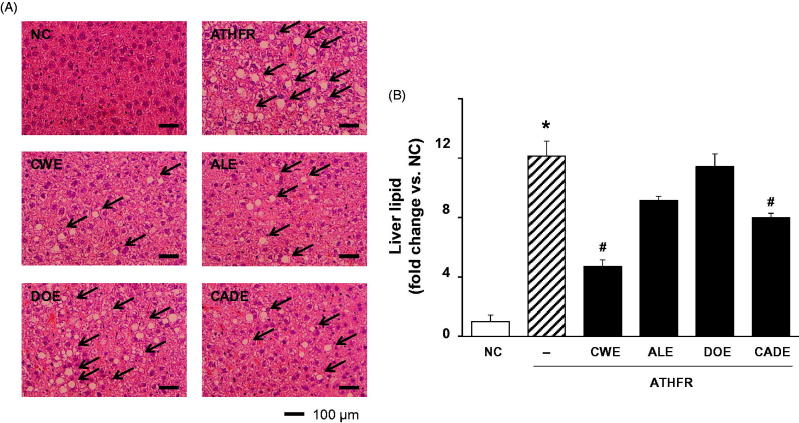
Effect of CWE, ALE, DOE and CADE on lipid contents in livers from ATHFR diet-fed mice. Mice were fed with normal chow diet or an ATHFR diet in the presence or absence of CWE, ALE, DOE and CADE for 8 weeks (each *n* = 6). At the end of the experiment, liver specimens were collected, and frozen liver sections were stained with H&E staining. Omega-3 and simvastatin were used as controls of nutritional alternative and lipid-lowering drug, respectively. Original magnification, ×200. Bar indicates 100 μm. *p < 0.05, significantly different from the NC group, #*p* < 0.05, significantly different from the ATHFR group. ATHFR: an atherogenic diet plus 10% fructose; CWE: *C. wilfordii* extract; ALE: *A. lappa* L. extract; DOE: *D. opposita* extract; CADE: mixture of CWE:ALE:DOE with ratio of 2:1:1; NC: untreated control.

**Figure 10.**
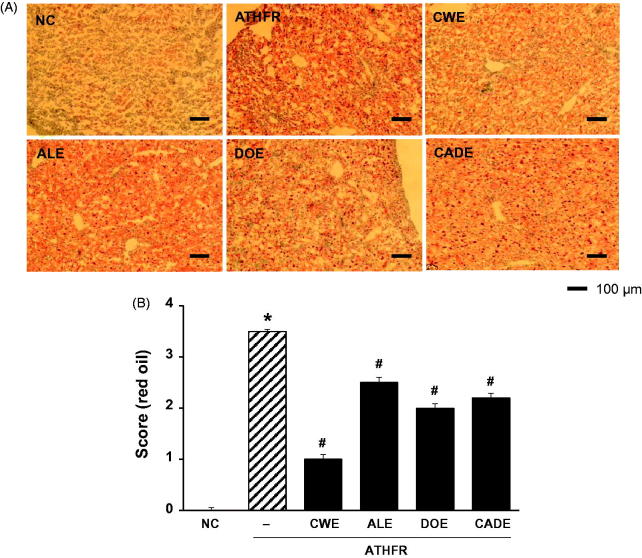
Effect of CWE, ALE, DOE and CADE on fat accumulation in livers from ATHFR diet-fed mice. Mice were fed with a normal chow diet or an ATHFR diet in the presence or absence of CWE, ALE, DOE or CADE for 8 weeks (each *n* = 6). At the end of the experiment, liver specimens were collected, and frozen liver sections were stained with H&E staining. Omega-3 and simvastatin were used as controls of nutritional alternative and lipid-lowering drug, respectively. Original magnification, ×100. Bar indicates 100 μm. *p < 0.05, significantly different from the NC group, #*p* < 0.05, significantly different from the ATHFR group. ATHFR: an atherogenic diet plus 10% fructose; CWE: *C. wilfordii* extract; ALE: *A. lappa* L. extract; DOE: *D. opposita* extract; CADE: mixture of CWE:ALE:DOE at a ratio of 2:1:1; NC: untreated control.

Liver damage may be associated with aberrant accumulation of fat in macrovesicles and microvesicles. ATHFR diet-fed mice had marked macrovesicular and microvesicular steatosis (Figure 9). These accumulations of fat in the liver were lessened by supplementation with CWE, ALE and CADE. When liver tissues were stained with oil red O-staining and fat accumulation was scored (Figure 10), we determined that high accumulation of fat in the liver tissue was attenuated by supplementation with CWE, ALE and DOE. The suppressive potential of CADE supplementation on the liver fat accumulation was better than that of ALE but less than that of the CWE alone group. Omega-3 and Simv at the given doses were similar to that of the CWE alone group.

### Effect of CWE, ALE, DOE and CADE on protein expression of VCAM-1 in the liver

**Figure 11.**
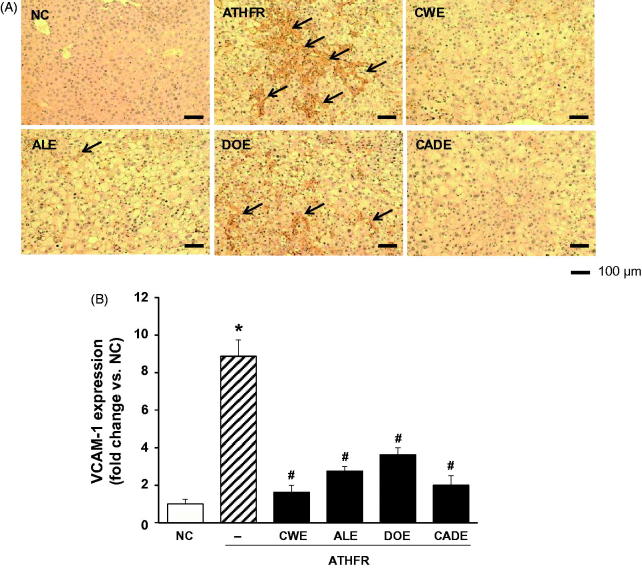
Effect of CWE, ALE, DOE and CADE on protein expression of VCAM-1 in livers from ATHFR diet-fed mice. Mice were fed with a normal chow diet or an ATHFR diet in the presence or absence of CWE, ALE, DOE or CADE for 8 weeks (each *n* = 6). At the end of the experiment, liver specimens were collected, and protein expression levels of VCAM-1 were detected in the frozen liver sections using immunohistochemical staining. Original magnification, ×100. Bar indicates 100 μm. *p < 0.05, significantly different from the NC group, #*p* < 0.05, significantly different from the ATHFR group. ATHFR: an atherogenic diet plus 10% fructose; CWE: *C. wilfordii* extract; ALE: *A. lappa* L. extract; DOE: *D. opposita* extract; CADE: mixture of CWE:ALE:DOE at a ratio of 2:1:1; NC: untreated control.

An increase in protein expression of VCAM-1 in tissue may reflect the presence of an inflammatory response. When liver protein expression levels of VCAM-1 were determined using an immunohistochemical method, ATHFR diet-fed mice showed a markedly increase protein expression level of VCAM-1 in the liver (Figure 11). Both omega-3 and Simv were also completely block the VCAM-1 expression in the liver (data not shown). Interestingly, CADE supplement blocked the VCAM-1 expression in the liver with comparable potency to CWE.

## Discussion

CADE, a mixture of CWE, ALE and DOE at a 2:1:1 ratio, was prepared based on the suppressive effect on the mRNA expression levels of VACM-1 and ICAM-1 in HASMC (Figure 1) and the inhibitory effects on adipogenesis in 3T3-L1 cells (Figure 2). While the lipid-lowering effect of CADE was less potent than that of CWE, supplementation with CADE suppressed inflammatory responses in the aorta even with relatively high lipid levels compared with that of CWE (Figures 6 and 7). In addition, liver function was better preserved by CADE supplementation than by CWE supplementation alone (Figure 8). As with CWE, supplementation with CADE was effective in suppressing VCAM-1 induction (Figure 11).

Multiple risk factors interact to damage the endothelium. Among them, elevated lipids are central to the development of atherosclerosis (Weintraub 2002). Numerous phytochemical compounds have been studied as potential tools to regulate glucose homeostasis, adipose tissue development, and inflammatory tone for treatment of diabetes, atherosclerosis, and metabolic syndromes, respectively. Natural products continue to be valuable sources for drug development (Harvey 2000). It has been suggested that lipid-lowering drugs such as statins cannot completely control blood lipid levels in all patients or even in high risk people for a variety of reasons. In the absence of appropriate lipid-lowering, vascular lesions and hepatic damage may arise due to the aberrantly high lipid levels, which, in turn, may result in pathological manifestations.

Synergy occurs if two or more herbal ingredients mutually enhance each other’s effect more significantly than the simple sum of these ingredients (Williamson 2001; Gilbert and Alves 2003; Ma et al. 2009). An extract of *C. wilfordii* Radix (CWE) was shown to ameliorate ATHFR-induced liver damage by suppressing up-regulation of COX-2, possibly through the action of NF-κB and p38 MAPK. In addition, CWE treatment improved blood lipid profiles and reduced fat accumulation in the liver (Jang et al. 2016). *A. lappa* extract was shown to ameliorate the high fat/cholesterol diet-induced vascular dysfunction by protecting vascular relaxation and suppression of vascular inflammation (Lee et al. 2012; Wang et al. 2016). As alternative medicines for treatment of atherosclerosis, herbal formulations are most beneficial if they exert protective actions against vascular and liver damage beyond their lipid-lowering activity. We hypothesized that a combination of CWE, ALE and DOE would have enhanced efficacy on the suppression ATHFR diet-fed induced vascular inflammation and liver damage using a reduced concentration of each extract. When CWE, ALE and DOE were combined at a 2:1:1 ratio (CADE), the suppressive effect of CADE on the mRNA expression levels of VCAM-1 and ICAM-1 was maximized in HASMC cells (Figure 1). The anti-lipogenetic effect of CADE was second to CWE alone. In general, the potential of extract will show dose-dependence before reaching the toxicity level. Considering that CADE contains half of the concentration of CWE used in the CWE alone studies, these suppressive effects on the expression of VCAM-1 and ICAM-1 and lipogenesis are acceptable. The bulk cultivation of *C. wilfordii* Radix, *A. lappa* and *D. opposita* is not an easy task, and the costs of production and isolation are high. Thus, reduction of the quantity of each component makes their use more economical without loss of therapeutic efficacy against ATHFR-mediated vascular and liver damage.

In ATHFR diet-fed mice, CWE at the given doses showed the strongest potential in suppressing increased tissue weight in both liver and spleen, lowering high-cholesterol (or TG), lessening aortic thickness, and reducing liver fat accumulation and impairment in liver function. The reduction of vascular and hepatic inflammatory responses seems to be closely associated with high efficient suppression of accumulation of blood T-C, LDL-C and TG (Figure 5), since the high status of these lipid levels can contribute to pathological changes in the vasculature and liver. Both ALE and DOE inhibited increases in these lipid levels with less efficacy than CWE; thus CADE could not markedly suppress the increase of lipid levels. It is unclear why simvastatin and omega-3 were not effective on the lowering of LDL-C and TG level in this experiment (Figure 5). Less effectiveness of simvastatin and omega-3 on lipid lowering in this experiment may be associated with insufficient duration of treatment, since lipid-lowering effect of statins may be, at least in part, associated with the stimulated excretion of cholesterol from the body (Schonewille et al. 2016). The action of CWE and DOE on the suppression of aortic thickening might share the same lipid-lowering, and, thus, CADE, which contains half the concentration of CWE and one quarter that of DOE, did not show an enhanced efficacy on the reduction of aortic thickness (Figure 6). The aortic expression of VCAM-1 and P-selectin were markedly suppressed by CADE supplementation. This action of CADE might not be associated with simple reduction of blood lipid levels, since the lipid-lowering effect of CADE was less than that of ALE or DOE alone. This action of CADE on the suppression of aortic expression of VACM-1 and P-selectin might be a synergistic effect from the combined actions of CWE, ALE and DOE. Impaired liver function, as assessed by GOT and GTP levels in ATHFR diet-fed mice, were markedly alleviated by CADE supplementation and was similar to the CWE alone group. This result is another example of the synergistic effect that occurred with the combination of CWE, ALE and DOE (Figure 8). The inhibitory effect of CADE on fat accumulation in the liver was not enhanced and may be associated with less reduction effect of CADE on lipid-lowering (Figures 9 and 10). In the liver, CADE showed a synergistic suppressive effect on the expression of VCAM-1 (Figure 11) since CADE was more potent than either ALE or DOE alone. The suppressive effect of CADE on the vascular inflammatory response and the alleviation of impairment of liver function and fat accumulation is an alternative for maintaining vascular and liver heath in high lipid conditions.

## Conclusions

In conclusion, CADE that is CWE, ALE and DOE combined at a 2:1:1 ratio showed a remarkable lipid-lowering effect as well as suppression of vascular and liver inflammatory response and impairment of liver function. These effects of CADE might not be associated with its enhanced lipid-lowering potential, but may rather be an unidentified synergistic effect occurred from combining CWE, ALE and DOE. Future extensive works will be necessary to elucidate effect of CADE to strengthen the lipid-lowering potential with protection against vascular and liver damage.
